# Temperature- and pH-Responsive Super-Absorbent Hydrogel Based on Grafted Cellulose and Capable of Heavy Metal Removal from Aqueous Solutions

**DOI:** 10.3390/gels9040296

**Published:** 2023-04-02

**Authors:** Hebat-Allah S. Tohamy, Mohamed El-Sakhawy, Beata Strachota, Adam Strachota, Ewa Pavlova, Silvia Mares Barbosa, Samir Kamel

**Affiliations:** 1 Cellulose & Paper Department, National Research Centre, 33 El-Bohouth St., Dokki, Giza 12622, Egypt; 2 Institute of Macromolecular Chemistry, Czech Academy of Sciences, Heyrovského nám. 2, 162 00 Praha, Czech Republic

**Keywords:** cellulose-based hydrogels, graft copolymerization, stimuli-sensitive hydrogels, Cr(VI) adsorption, swelling kinetics

## Abstract

In this work, we prepared highly swelling, stimuli-responsive hydrogels capable of the highly efficient adsorption of inorganic pollutants. The hydrogels were based on hydroxypropyl methyl cellulose (HPMC) grafted with acrylamide (AM) and 3-sulfopropyl acrylate (SPA) and were synthesized via the growth (radical polymerization) of the grafted copolymer chains on HPMC, which was activated by radical oxidation. These grafted structures were crosslinked to an infinite network by a small amount of di-vinyl comonomer. HPMC was chosen as a cheap hydrophilic and naturally sourced polymer backbone, while AM and SPA were employed to preferentially bond coordinating and cationic inorganic pollutants, respectively. All the gels displayed a pronounced elastic character, as well as considerably high values of stress at break (several hundred %). The gel with the highest fraction of the ionic comonomer SPA (with an AM/SPA ratio = 0.5) displayed the highest equilibrium swelling ratio (12,100%), the highest volume response to temperature and pH, and the fastest swelling kinetics, but also the lowest modulus. The other gels (with AM/SPA = 1 and 2) displayed several times higher moduli but more modest pH responses and only very modest temperature sensitivity. Cr(VI) adsorption tests indicated that the prepared hydrogels removed this species from water very efficiently: between 90 and 96% in one step. The hydrogels with AM/SPA ratios of 0.5 and 1 appeared to be promising regenerable (via pH) materials for repeated Cr(VI) adsorption.

## 1. Introduction

Egypt is one of the most water-deficient regions in the world due to the hot and arid climate; desert landscape; scarce freshwater sources; continued expansion of urbanized areas; progress of industrialization; and, hence, man-made contamination of drinking water [1]. Industrial wastewater often needs special cleaning procedures because of the presence of metal compounds. Among them, Cr(VI) poses a special threat to human health because of its high toxicity [2]. It is a prominent contaminant, because in industry, Cr(VI) compounds are used in numerous applications such as galvanization processes and anodic metal surface passivation, as pigments, and for the preservation of wood [2].

In water-deficient countries, the purification of contaminated industrial wastewater is an essential issue and is being intensely explored [3,4,5]. In this context, the present work was dedicated to stimuli-responsive hydrogels, which might be capable of the cyclic adsorption/desorption of toxic metal compounds, and the highly toxic Cr(VI) was selected as the substance to be test-adsorbed.

Historically, adsorption on activated carbon has been the most widely used water purification technique [6]. However, adsorption on carbon has a low efficiency if metals and inorganic pollutants have to be removed, in contrast to the highly efficient adsorption of organic compounds on carbon. Attempts have been made to increase the adsorption-able surface of activated carbon, as well as its selectivity towards inorganic pollutants, using modification and impregnation techniques [7]. As alternative and promising adsorber systems, stimuli-responsive polymers have started to be investigated for metal removal applications, and they are able to achieve a high selectivity and removal efficiency for inorganic pollutants, while the technique for the adsorption and regeneration processes is fairly simple [3,4,8]. Such promising smart polymers have been tested as recyclable separation materials in the form of solutions, particles, fibers, mats, and membranes [9,10,11,12,13,14,15,16,17,18,19].

Stimuli-sensitive ‘smart’ hydrogels most typically respond to either temperature or pH variations by a distinct change in swelling degree: they swell (and thus expand) or deswell (shrink) as a result of their stimulus-induced transition from a hydrophilic to a hydrophobic state, or vice versa. In the case of a high ratio of maximum to minimum swelling degree, these materials are attractive for potential use as recyclable super-absorbents [14,16]. A porous morphology is advantageous for adsorption applications because of the possibility of the rapid transport of water into the gel upon swelling and the high specific surface area of the adsorbing material. This makes possible rapid pollutant adsorption. The modification of the composition of the hydrogel by suitable comonomers can significantly improve its adsorption efficiency and/or increase its swelling ability [3].

From an economical point of view, it is attractive to employ non-expensive natural materials for the eventual mass-production of adsorber hydrogels for use in water treatment [9]. Cellulose is the most abundant natural polymer on Earth, so it is inexpensive, and it can also be modified to a water-soluble derivative, which in turn can be used as a major component of smart hydrogels [20]. Hydroxypropylmethyl cellulose (HPMC), obtained by partial etherification and hydroxypropylation, is one such water-soluble derivative. HPMC is widely used in many products as a polymer matrix due to its biocompatibility and biodegradability, and it has hence inspired numerous scientific studies into new applications [10,21].

The main drawbacks of simple chemically crosslinked non-porous hydrogels are their poor mechanical properties and slow response to external stimuli (in the case of smart derivatives). One way to improve the mechanical properties of hydrogels is to combine polysaccharide materials and synthetic polymers through graft copolymerization [18]. Of interest as a grafted component is polyacrylamide (or its derivatives), which was shown to possess good adsorption properties [22]. The embedding of intercalated polysaccharide (starch) into a poly(N-isopropyl acrylamide) hydrogel has also been used to improve the mechanical properties of super-fast responsive gels [23]. In addition, in [23], the intercalation of polysaccharides into a temperature-sensitive hydrogel was shown to considerably accelerate its deswelling process.

The main objective of the current work was to synthesize pH- and thermo-responsive hydrogels prepared from naturally sourced hydroxylpropylmethyl cellulose, onto which copolymer chains were grown by the graft polymerization of acrylamide (AM) and 3-sulfopropyl acrylate potassium salt (SPA) for the purpose of obtaining a recyclable smart gel that could adsorb inorganic pollutants such as Cr(VI), with a potential application in water treatment. The designed structure was expected to lend the gels good mechanical properties and enable the rapid adsorption of pollutants.

The role of the AM monomer was to adsorb (via coordination bonding as a neutral ligand) pollutants such as transition-metal cations [22,24], as well as to act as a hydrophilic component of the gel. On the other hand, SPA is a pH-stable anionic comonomer that was expected to bind metal cations via electrostatic interaction. We also predicted that SPA would greatly increase the hydrophilicity of the gel, which was to be tuned by the AM/SPA ratio. The structure of the whole hydrogel was fixed by the divinyl crosslinker *N,N’*-methylenebis(acrylamide) (MBA), which crosslinked the chains grafted on neighboring HPMC units. The authors acquired experience with such grafted and additionally crosslinked structures in their recent work, e.g., [25]. The synthesis technique employed to graft polyacrylates onto polysaccharides, namely the use of a strong oxidant for generating radicals on the polysaccharide, is widely reported in the literature, e.g., [26,27,28], with many variations.

## 2. Results and Discussion

**Scheme 1:**
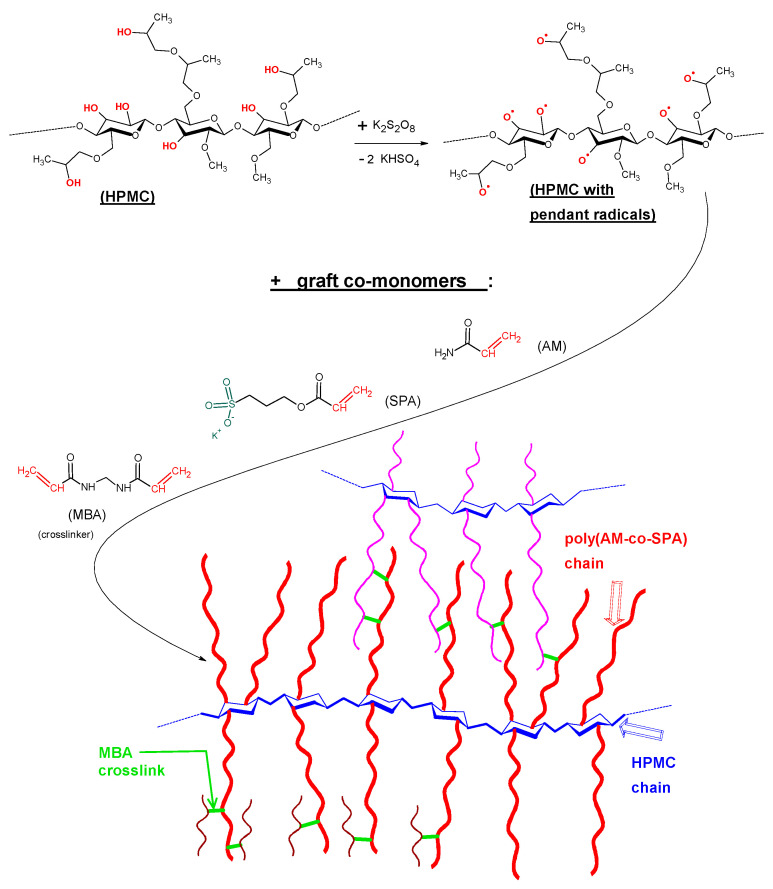
Synthesis of HPMC-g-poly(AM-co-SPA) hydrogels: idealized structures.

The stimuli-responsive hydrogel HPMC-g-poly(AM-co-SPA) was synthesized according to Scheme 1. It was designed to be tested as a recyclable superabsorbent smart gel for potential application in wastewater treatment, i.e., for the removal of inorganic contaminants such as the highly toxic Cr(VI).

HPMC-g-poly(AM-co-SPA) was obtained (see Scheme 1) via the growth of grafted copolymer chains composed of acrylamide (AM) and 3-Sulfopropyl acrylate potassium salt (SPA) units on hydroxypropyl methylcellulose chains (HPMC). These grafted structures were crosslinked to an infinite network by a small amount of a third comonomer, *N,N′-*methylenebis(acrylamide) (MBA, a divinyl monomer), which interconnected the grafted chains growing from neighboring HPMC macromolecules. The idealized final structure of the synthesized hydrogels is shown in Scheme 1. HPMC served as a cheap, naturally sourced and biodegradable hydrophilic gel component, while the AM comonomer had to act as a coordinating adsorber of transition metals, and SPA was incorporated in order to enable the electrostatic fixation of cations and tune the hydrophilicity of the whole gel. The main comonomers, AM and SPA, were used in three different ratios (A/S = 0.5, 1, and 2) in order to optimize the gel for its potential applications. The content of the chemical components was: 25 wt.% HPMC + 75 wt.% grafted poly(AM-co-SPA). The swelling after complete synthesis was between 750% and 1680% (if progressing from A/S = 2 to 0.5).

The hydrogel synthesis consisted of two steps (see Scheme 1): in the first step, potassium persulfate (KPS), a strong oxidant, was added to generate radicals on suitable sites of the dissolved HPMC macromolecules. After this ‘activation’ of HPMC was complete, the comonomers AM, SPA, and MBA (a small amount of crosslinker) were added in the second step, which consisted of the radical graft polymerization of these comonomers, starting from the radical sites on HPMC. The synthesis procedure was analogous to that in the authors’ previous works [25].

The synthesis technique of grafting hydrophilic polymers onto a polysaccharide backbone by generating radicals on this backbone in the first step using strong oxidants such as KPS, followed by vinyl graft polymerization in the second, has been described as a successful route in the literature, e.g., [26] (alginate backbone, acrylic acid as a graft monomer, and ammonium persulfate as a radical oxidant). This method has also been applied more widely for the grafting of collagen with poly(acrylic acid), as described in [27] (also using ammonium persulfate as oxidant). Recently, the authors of this paper successfully applied this method to the grafting of sodium carboxymethyl cellulose with acrylamide (doped with SPA comonomer) using KPS as an oxidant [25].

### 2.1. Chemical Characterization of the HPMC-g-Poly(AM-co-SPA) Hydrogels

#### 2.1.1. Porous Morphology and Elemental Analysis of Freeze-Dried Hydrogels

**Figure 1:**
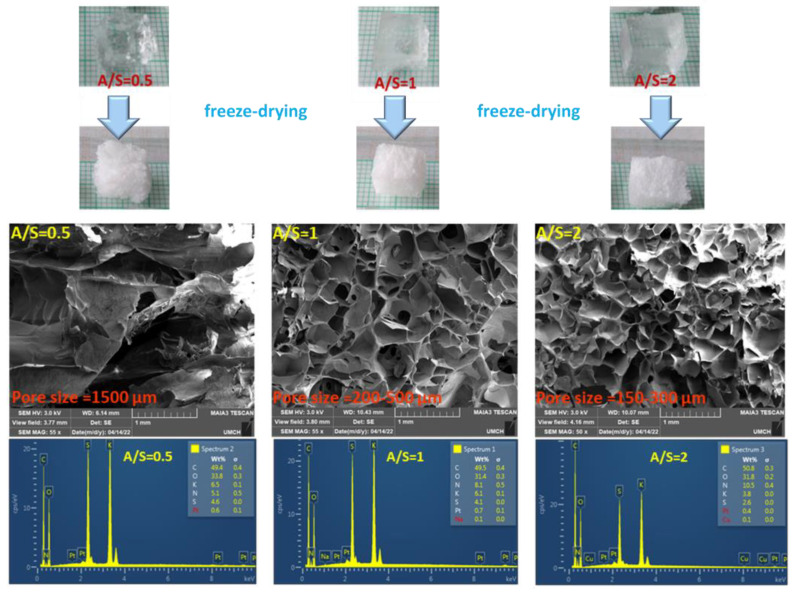
The prepared HPMC-g-poly(AM-co-SPA) hydrogels before and after freeze-drying; SEM images of their porous morphology; and the EDX elemental analysis of these hydrogels.

The synthesized products were obtained as highly (AM: SPA = 1:2 ≡ “A/S = 0.5”) to fairly (A/S = 1 and 2) transparent bulk hydrogels (see Figure 1 top).

For the purpose of the swelling and adsorption tests, the gels were freeze-dried in order to achieve an interconnected porous morphology (white samples in the second row in Figure 1). This morphology was advantageous for adsorption applications because of the possibility of rapid water transport into the gel upon swelling and the high specific surface area offered by the pore walls. The third row in Figure 1 shows the porous morphologies of our products, as observed by SEM. It can be seen that the final freeze-dried hydrogels were hollow sponge materials with rounded pores. The pore sizes were ca. 1500 µm (1.5 mm), 200–500, and 150–300 µm for the samples with A/S = 1, A/S = 0.5, and A/S = 2, respectively. Hence, they markedly decreased with an increasing A/S ratio. This result could be correlated with the stiffness of the gels: The gel with A/S = 2 was the stiffest, and hence the pores generated by freeze-drying were the smallest. The sample with A/S = 0.5 contained the largest amount of the ionic and highly hydrophilic comonomer SPA, displayed the highest swelling degree prior to freeze-drying, and was the softest. The growth of ice crystals, which occurred in the initial stage of the freeze-drying process, met the smallest hindrance in the softest gel (which additionally was water-rich), and hence the largest crystals were generated in this material. The crystals subsequently sublimated and left behind the largest pores in A/S = 0.5.

The EDX elemental analyses (Figure 1, fourth row) confirmed the content of the hetero-elements from AM (nitrogen) and SPA (potassium and sulfur). The content of these elements, as expected, shifted with the changing A/S ratios.

#### 2.1.2. FTIR Analysis of Hydrogen Bonding

**Figure 2:**
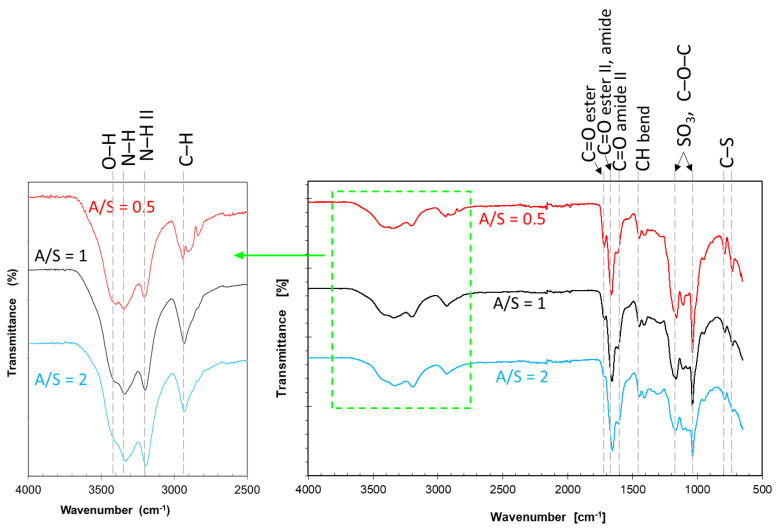
FTIR spectra of HPMC-g-poly(AM-co-SPA) hydrogels.

In order to evaluate the interactions between the components of the synthesized grafted hydrogels, the specimens of the gels with graft comonomer ratios of A/S = 1, A/S = 0.5, and A/S = 2 were dried and analyzed by FTIR spectroscopy (see Figure 2). The assignment of the most important peaks is shown in Figure 2.

The spectra were overlaid after being calibrated to possess the same intensity of the carbonyl peak at 1660 cm^−1^. Changes in hydrogen bonding would lead to changes in the peak position in the OH/NH stretching region (3600–3200 cm^−1^) and in the carbonyl region (1750–1580 cm^−1^). Generally, it was observed that the change in the ratio of the comonomers AM and SPA caused only very slight changes in hydrogen bonding, which affected only the AM units, and the highly visible changes in the spectra reflected the changes in the intensity of the characteristic signals of the grafted comonomers AM and SPA.

Hydrogen bonding analysis: In the OH/NH stretching region (see zoomed-in image in Figure 2), the broad peaks, which were relatively structured, possessed nearly identical intensities and areas if superimposed (not shown here). However, differences could be seen in the relative prominence of their components: The two maxima associated with amido NH_2_ groups (3340 and 3200 cm^−1^), as expected, were most prominent relative to the OH signal at ca. 3406 cm^−1^ (which originated from HPMC) at A/S = 2, while their contribution was most modest at A/S = 0.5. The positions of the local maxima of the broad OH/NH stretching peak practically did not change, but a slight systematic shift in the NH peaks could nevertheless be observed from 3348 cm^−1^ (A/S = 0.5) down to 3338 cm^−1^ (A/S = 2), which suggested an increase in the strength of hydrogen bonding. When the NH peaks were used to calculate the mean hydrogen bond strength (MHBS) according to [3,13,29] (see results in Table 1), very similar values were obtained (0.96–0.98), which differed within the error margin. The most intense carbonyl peak (associated with both ester and amido C=O groups, and hence with both comonomers) also shifted slightly from 1661 cm^−1^ (A/S = 0.5) down to 1654 (A/S = 2). This trend also indicated a somewhat stronger hydrogen bonding at higher contents of the AM monomer. Due to the structure of the copolymeric hydrogels, this seemed to be mainly H-bonding between AM units, which possess both active protons and electron pair donors (O). The smaller carbonyl peak at 1718 cm^−1^, which was associated exclusively with ester groups in the SPA comonomer, decreased as expected with an increasing A/S ratio. Another small carbonyl peak, namely that at 1612 cm^−1^, was characteristic of the amido groups, and it increased (and simultaneously slightly downshifted) with the A/S ratio (increase in H-bonding).

Characteristic peaks of SPA: Between 1260 and 966 cm^−1^, there was a group of peaks associated with the SPA comonomer, which accordingly decreased with a rising A/S ratio. The main peaks were at 1169 cm^−1^ (SO_3__I and C–O–C_ester_I) and 1037 cm^−1^ (COC_II with a recognizable underlying broader SO_3__II peak centered at ca. 1029 cm^−1^). The peaks at 790 and 730 cm^−1^ were associated with C–S stretching in the SPA units. The position of no SPA peak showed even a slight shift with a change in the A/S ratio.

#### 2.1.3. Thermal Stability (TGA)

**Figure 3:**
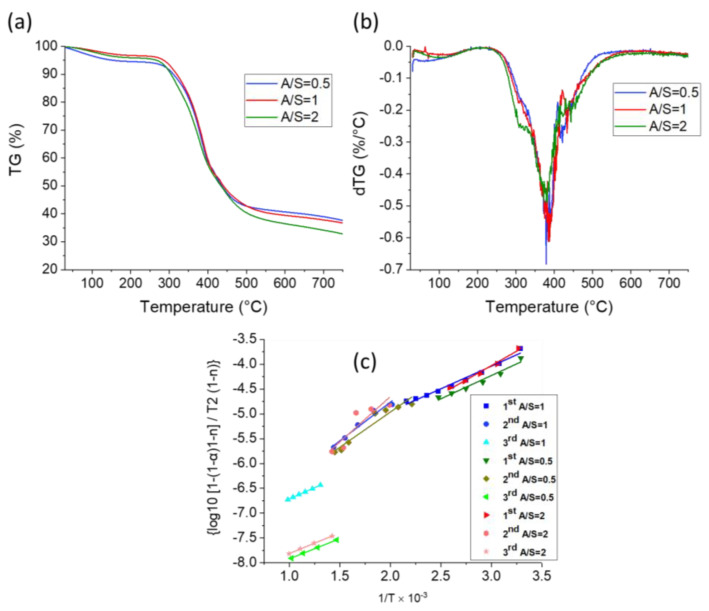
(**a**) TGA and (**b**) DTG of A/S = 1, A/S = 0.5, and A/S = 2; (**c**) their thermal kinetics.

In order to assess their relative long-term stability, as well as the segmental mobility in the synthesized copolymeric hydrogels, these products were subjected to thermogravimetric analyses (TGAs) in nitrogen. The TGA traces as well as their derivatives (DTG curves) are shown in Figure 3a,b, respectively, while the data obtained by their analysis are summarized in Table 2. The analysis of the kinetics of the observed decomposition processes is presented in Figure 3c. Generally, it can be observed that the copolymeric products displayed similar behavior, but that a higher SPA content led to a slightly lower stability and to a higher segmental mobility. All the freeze-dried hydrogels also released some residual water in the TGA tests. The highest fraction was found in the gel with the highest amount of the highly hydrophilic ionic comonomer SPA (A/S = 0.5).

The TGA curves of A/S = 1, A/S = 0.5, and A/S = 2 revealed three decomposition steps with a mass loss (ML%) of 57.14, 62.43, and 67.34%, respectively, at 800 °C, ascribed to the non-volatile contents.

The first ML stage was in the range of 30.5–168.2, 29.9–149.7, and 32.6–172.6 °C, with maximum temperatures of 84.3, 74.3, and 78.2 °C, and with an average ML of 3.53, 3.65, and 2.89%, respectively, and was assigned to moisture and/or residual water content.

The second ML stage was between 222.6 and 416.5, 179.0 and 400.2, and 229.6 and 411.6 °C, with maximum temperatures of 384.7, 379.1, and 380.8 °C, and with an average ML of 42.3, 36.5, and 41.4%, respectively. This ML stage was ascribed to the pyrolysis degradation of the polymer chains (especially of the flexible grafted chains).

The third ML stage was between 491.7 and 752.0, 408.9 and 759.7, and 429.7 and 759.7 °C, with maximum temperatures of 433.3, 421.2, and 443.5 °C, and with an average ML of 14.6, 21.2, and 21.9%, respectively. This stage corresponded to the decomposition of the carbonaceous pyrolysis residues to low-molecular-mass volatile products [3].

The above-mentioned temperature ranges of the mass loss (ML) are denoted as 1st, 2nd, and 3rd in Table 2 and in the analyses in Figure 3c.

When the TGA (DTGA) data were evaluated using the Coats–Redfern approach (see fits in Figure 3c), the thermodynamic and kinetic parameters of the individual decomposition processes were obtained (see Table 2), namely the changes in enthalpy (∆*H*), entropy (∆*S*), and free energy (∆*G*), as well as the kinetic magnitudes (activation energy (*E*a); frequency factor *A*; and the order of degradation reaction (*n*), which was found by fitting, using the smallest square method). The correlation coefficients (R^2^) for the fitting of *n* and the resulting standard error values (SE) are also provided in Table 2. Details of the kinetics evaluation can be found in the Materials and Methods section.

The high activation energy (*E*a) observed for A/S = 2 (*E* = 73.10 kJ mol^−1^) illustrated its higher thermal stability compared to A/S = 1 and A/S = 0.5 (*E* = 64.44 and 60.72 kJ mol^−1^).

Finally, the TGA results implied that the mobility of the polymer segments in A/S = 2 was suppressed in comparison to the other samples, which resulted in delayed polymer degradation [3,8,13]. This could be attributed to the H-bonding, which was found to be strongest in A/S = 2 by means of FTIR spectroscopy (MHBS evaluation).

### 2.2. Swelling Behavior

#### 2.2.1. Kinetics of Swelling of Dried Porous Gels

**Figure 4:**
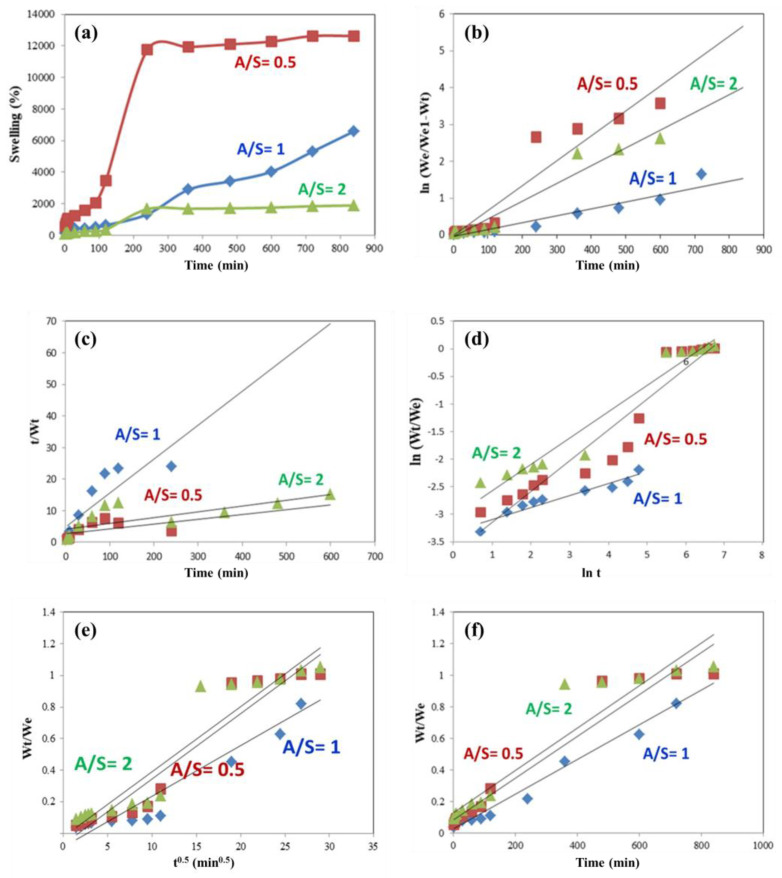
(**a**) Swelling behavior, (**b**) pseudo-first-order, (**c**) pseudo-second-order, (**d**) Peppas and Peppas, (**e**) Fick’s mechanism, (**f**) Peppas and Franson method of HPMC-g-poly(AM-co-SPA) hydrogels.

In view of the envisaged potential application as super-absorber materials, the swelling kinetics of the HPMC-g-poly(AM-co-SPA) hydrogels was of great importance. It was investigated to the maximum extent, starting from the freeze-dried state. Freeze-drying was applied in order to generate porosity in the gels and thus to enable their relatively rapid water uptake combined with the adsorption of inorganic compounds onto a large pore wall area. The observed kinetics, as well as their fitting with several kinetics models, are illustrated in Figure 4. Generally, the results could be summarized as follows: 3 to 5 h were needed to achieve full swelling equilibrium (starting from the fully dried state), which appeared to be a reasonable duration in view of the time needed for the efficient adsorption of pollutants from the solution that found itself in the pores of the hydrogels (1 to 2 h), as will be discussed further below. The gel with the highest content of the hydrophilic SPA comonomer, A/S = 0.5, displayed the fastest kinetics in addition to the highest swelling.

In order to assess the effect of the grafted copolymer structure of the hydrogels on their swelling behavior, a comparison with similar simpler systems was of interest. In a previous work [23], the authors characterized as a reference sample a strongly temperature-responsive variety of the simple AM gel, namely poly(N-isopropylacrylamide) (PNIPAm) crosslinked by MBA and containing no further additives or comonomers. In its non-porous bulk and partly swollen form, it needed ca. 2 days to complete its re-swelling response to a temperature stimulus. The re-swelling of a wet porous variety of PNIPAm crosslinked by MBA needed between 2 h and 1 day, depending on the precise porous structure [30]. However, from the dried state, long re-swelling times (ca. 1 day) were needed for non-modified porous PNIPAm [31]. In this comparison, the grafted structure of the studied gels appeared to clearly support a faster re-swelling (3–5 h).

Figure 4a displays the curves of water uptake by the initially dry hydrogels. A remarkable feature visible in the curves of the swelling ratio versus time was that initially, the swelling ratio increased slowly; thereafter more rapidly; followed by reaching a maximum constant swelling. This was the case of the lower-swelling A/S = 2 and of the higher-swelling A/S = 0.5. The gel A/S = 1 displayed an intermediate swelling between the two, and after a temporary stabilization it displayed a second region of accelerated swelling (instead of an equilibrium plateau) for long contact times. The intermediate course and shape of the curve of the gel with A/S = 1 seemed to be the result of the balanced effects of the AM and SPA comonomer. AM is a considerably hydrophilic monomer, while SPA is an ionic monomer that is even more hydrophilic [32]. The second region of accelerated swelling in A/S = 1 at t > 500 min might have been connected with the disruption of hydrogen bonding between AM groups in order to make possible a more un-coiled and swollen chain conformation, in which the SPA units were more hydrated. After 6–8 h at pH 7.0 (and at room temperature), the swelling achieved by the A/S= 0.5, A/S= 1, and A/S= 2 gels was 12097, 4028, and 1887%, respectively.

The fits of several kinetics models of the swelling process are depicted in Figure 4b–f (the parameters obtained from the fits are listed in Table 3) and discussed below.

Comparing the first- and second-order kinetics models, the pseudo-first-order kinetic more closely predicted the swelling kinetics in all three hydrogel samples. It was observed that the fitted rate constant *K*_1_ produced the best coefficients of correlation (R^2^: their values were higher and closer to 1, see Table 3) for all the tested samples when compared with the second-order kinetics fit. At the same time, the values of the equilibrium absorption quantity *W*_e1_ obtained from the pseudo-first-order model were well-suited for depicting the swelling kinetics of A/S = 1, A/S = 0.5, and A/S = 2. Hence, all these gels could be described as exhibiting both chemisorption and physisorption (Figure 4b,c) [33].

The value of the kinetics exponent *n* in the Peppas and Peppas model of water-transport in hydrogel networks (fit in Figure 4d) provided an indication of the transport mechanism. Representative water transport curves and fits for the A/S = 1, A/S = 0.5, and A/S = 2 hydrogels are shown in Figure 4d. For A/S = 1, A/S = 0.5, and A/S = 2, the obtained values of *n* = 0.2, 0.5, and 0.4, respectively, indicated Fick-type diffusion (the *n* values were below 0.6, i.e., fractional values) [34].

When the kinetics data were fitted with the equation describing Fick’s mechanism (fit in Figure 4e), the values of the diffusion coefficient *D* for the prepared HPMC-g-poly(AM-co-SPA) hydrogels improved with an increasing SPA content in the networks (i.e., A/S = 0.5) due to the resulting higher swelling capacity [35].

The fit according to the Peppas and Franson theory yielded the water penetration velocity υ for the HPMC-g-poly(AM-co-SPA) gels. Similarly to *D*, the water penetration velocity υ also increased with an increasing content of SPA, which led to an increased swelling capacity (highest at A/S = 0.5).

#### 2.2.2. pH-Sensitive Behavior

**Figure 5:**
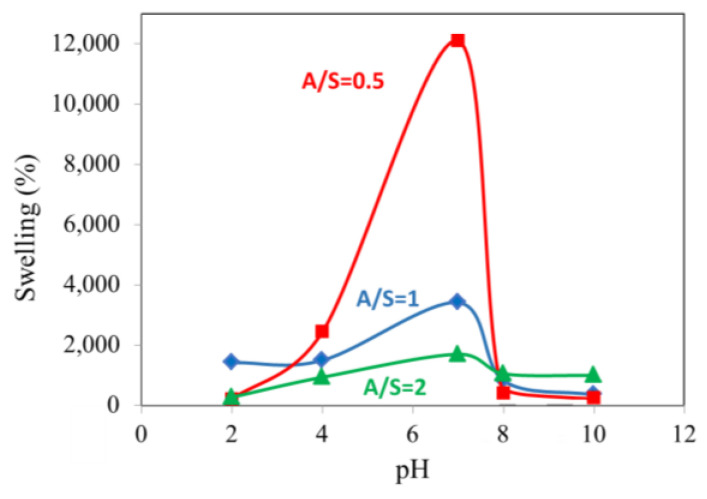
pH-responsive behavior of HPMC-g-poly(AM-co-SPA) hydrogels.

The pH-sensitivity of the prepared HPMC-g-poly(AM-co-SPA) hydrogels was of great importance for their potential application as regenerable adsorbers, as the cationic or anionic inorganic pollutants would first be adsorbed, before being released later by a pH stimulus, which would simultaneously induce the gel to shrink. The pH-responsive behavior of the hydrogels with comonomer ratios of A/S = 0.5, A/S = 1, and A/S = 2 in the pH range of 2.0 to 10.0 is compared in Figure 5. All the samples were clearly found to be pH-sensitive: they exhibited a markedly lower swelling ratio in the buffers compared to in distilled water (pH = 7). In the case of A/S = 2, the swelling response to pH-buffers was modest, but even in this case, the relative shrinking at pH = 2 was vast.

The pH-responsivity could be attributed entirely to the ionic strength effect: The SPA comonomer, which generated the pH response, was a pH-stable ionic unit, whose main role in the gels was to adsorb metal cations. It also greatly raised the hydrophilicity of the gels in pure water. However, the addition of relatively concentrated polyelectrolytes, such as acidic or alkaline buffer solutions (or other strong acids or bases), in both cases caused ionic aggregation (electrostatic crosslinking), and hence deswelling. Ionic strength effects have been widely discussed in the literature for different types of polyelectrolytes [36,37,38].

#### 2.2.3. Thermo-Sensitive Behavior

**Figure 6:**
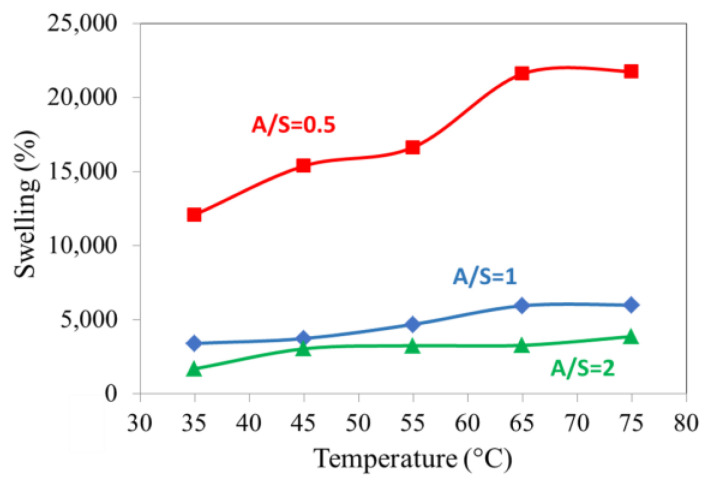
Temperature-responsive behavior of HPMC-g-poly(AM-co-SPA) hydrogels.

Temperature-sensitivity is another smart property that could be useful for the application of the studied HPMC-g-poly(AM-co-SPA) hydrogels as regenerable adsorbers of inorganic compounds. A vast water uptake or release in response to temperature could be a useful step in the work cycle, besides the above-mentioned pH-sensitivity. Figure 6 shows the temperature-dependent swelling for all the studied gels.

It can be observed in Figure 6 that all the studied hydrogels showed a continuous increase in swelling as the temperature increased from 35 to 75 °C. There were no distinct steps in the values of swelling. This pattern of temperature-dependence is characteristic of simple hydrophilic materials [39]. It can also be noted that for all the samples, the swelling approximately doubled between 35 and 75 °C, so that their volume expansion or shrinkage in response to a larger temperature stimulus was approximately the same. On the other hand, in absolute terms, the swelling of A/S = 1 was somewhat higher than that of A/S = 2, while the swelling of A/S = 0.5 was much higher at all temperatures than in the other gels as a result of the dominant effect of the hydrophilic SPA comonomer. At 75 °C, the equilibrium swelling was 21,800, 6000, and 3900% for comonomer ratios of A/S = 0.5, A/S = 1, and A/S = 2, respectively.

### 2.3. Mechanical and Viscoelastic Properties

The mechanical properties were of key importance for the practical application of the prepared hydrogels, especially for their manipulation during the adsorber work cycle. Regarding the porosity of the gels, their mechanical properties could vary widely according to the parameters of the freeze-drying process, especially due to swelling prior to freeze-drying (and eventual low-molecular additives), which could lead to different pore sizes and pore wall thicknesses. For this reason, the basic mechanical properties of the hydrogels were tested in the bulk non-porous state, obtained just after synthesis, as future pore wall material. Generally, it was found that all the gels displayed a pronounced elastic character, as well as considerably high values of stress at break (several hundred %). The APS comonomer was found to cause less efficient crosslinking and more strongly damping viscoelastic behavior.

#### 2.3.1. Gel stiffness and Crosslinking Density (after Synthesis)

**Figure 7:**
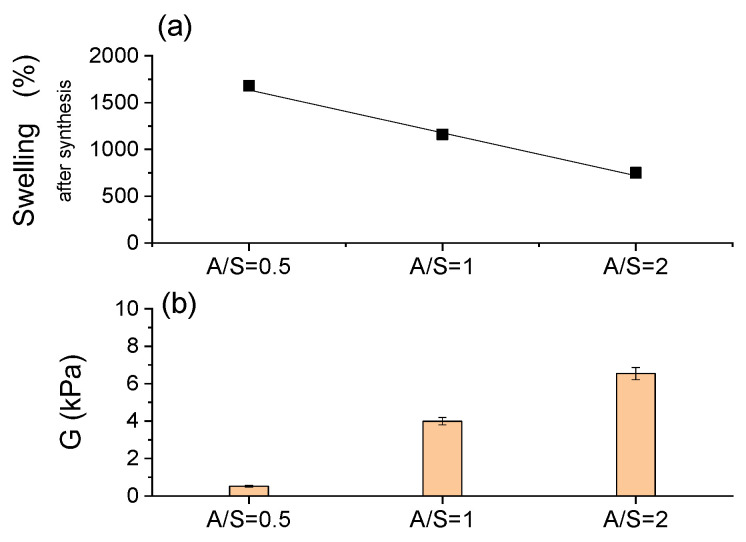
Swelling ratio (Q) (**a**) and shear moduli (**b**) of HPMC-g-poly(AM-co-SPA) hydrogels after synthesis.

Figure 7 shows the swelling values and the moduli of the A/S = 0.5, A/S = 1, and A/S = 2 hydrogels just after synthesis. The sample with the largest amount of hydrophilic SPA (i.e., A/S = 0.5) was exceptionally soft and showed the highest swelling (1680%), as well as the smallest modulus (520 Pa). In the case of the samples A/S = 1 and A/S = 2, the values were G = 3993 Pa/1160% and G = 6540 Pa/750%, respectively. The swelling values indicated that the hydrophilic comonomer SPA caused increased water uptake during the brief washing of the gels after synthesis. It could be seen that in the studied series, the swelling of the gels increased 2.24 times when advancing from A/S = 2 to A/S = 0.5. If the crosslinking density was the same in all gels, the modulus would be 2.24 times lower in the gel with A/S = 0.5. We observed a decrease by a factor of 12.6. This in turn suggested that if the gels were compared at the same swelling value, the modulus (and hence the crosslinking density) of A/S = 0.5 would still be 5.6 times smaller. Some disproportion in the ratios of the swelling values and moduli was also observed for the pair A/S = 1 and A/S = 2, but this disproportion was much smaller (the modulus decreased by 1.25 times more than what was expected from the swelling ratios). The comparison indicated that the hydrophilic SPA comonomer lowered the covalent crosslinking density in the hydrogels if it was incorporated. This could be attributed to the hydrophilic effect of the ionic sulfate group, which might favor solvation at the cost of entanglements during the formation of the network structure.

The theory of rubber elasticity yields a relation between the elastic modulus and the crosslinking density characterized by the number of elastically active chains. The magnitude of the concentration of the elastically active chains c(EAC) was used to characterize the crosslink density. The shear modulus G was used to calculate the c(EAC) via the formula G = c(EAC) R T, where R is the universal gas constant, and T is the temperature in Kelvin. The c(EAC) values for the samples A/S = 0.5, A/S = 1, and A/S = 2 were found to be 0.2, 1.6, and 2.6 mol/m^3^, respectively, at the swelling degrees after synthesis.

#### 2.3.2. Viscoelastic Properties and Deformation Limits

Because the viscoelastic properties could directly reflect the strength of chain entanglement and the branching extent of the grafted cellulose, dynamic rheology was an efficient way to assess the elastic and flow properties of the prepared hydrogels. The storage modulus (G’) and loss modulus (G”) provide information on the stress response of materials. G’ measures the energy that a material stores during a deformation cycle and represents the material’s elastic behavior. On the other hand, G” describes the energy dissipated and lost as heat during the shear cycle. In the A/S= 0.5, A/S= 1, and A/S= 2 hydrogels, over the whole frequency range, G’ and G” were constant, and the moduli increased with an increase in AM content. In the softest gel, where A/S = 0.5, the modulus G” displayed a weak frequency-dependence, but its curve was very flat.

**Figure 8:**
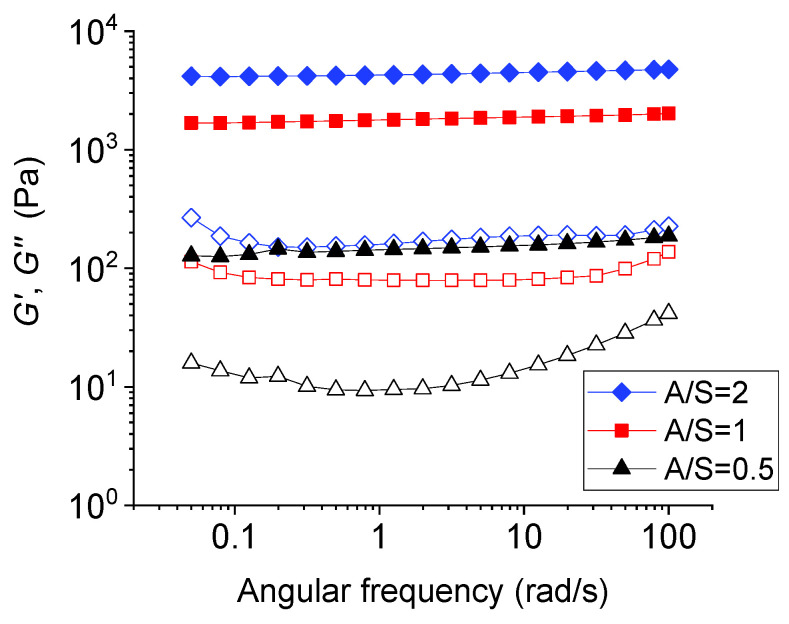
Frequency-dependence of storage shear modulus (G′) and the loss shear modulus (G″), as observed in oscillatory rheological investigations. Data points in the graph: G′—filled symbols; G″—open symbols.

Both the G’ and G” moduli displayed a similar trend to that of the static moduli discussed above. The AM chains favored a higher number of permanent crosslinks (covalent and possibly also entanglements), which might have been partly helped by the observed higher tendency of AM toward hydrogen bonding. The developed hydrogels behaved predominantly elastically because G’ was always larger than the G” of the corresponding hydrogel at any frequency (Figure 8) [40]. The G’/G” ratios differed between the prepared gels, and G” was relatively large, which is typical for non-ideal elastomers displaying a high solid-state viscosity. These large G” values indicated considerable mechanical damping in the gels. The damping was the highest (i.e., G’/G” was the smallest) in the softest gel with the least efficient crosslinking (A/S = 0.5), in spite of dilution by high swelling. This indicated a high number of dangling chains (grafted chains that were not crosslinked), which increased the molecular friction (energy dissipation) upon deformation.

**Figure 9:**
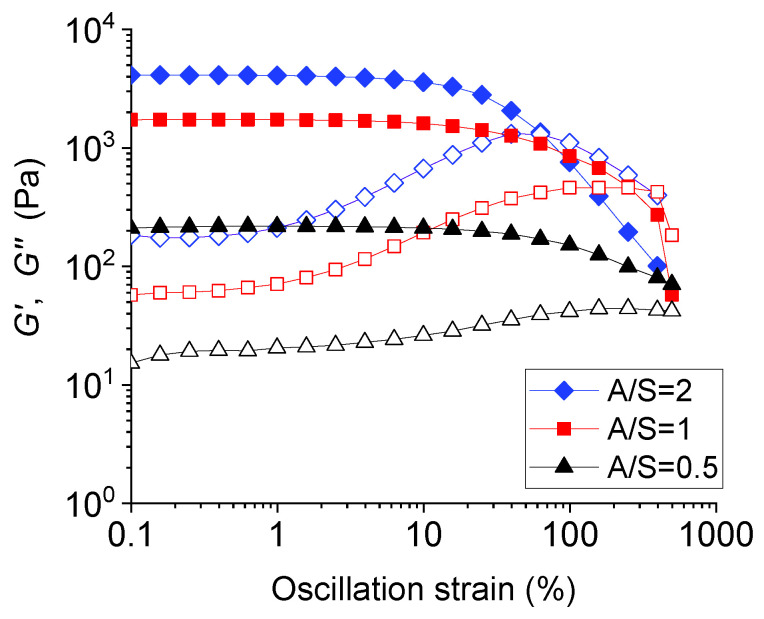
Storage modulus (G′) and loss modulus (G′′) vs. strain of HPMC-g-poly(AM-co-SPA) hydrogels. Data points in the graph: G′—filled symbols; G″—open symbols.

In order to evaluate the limits of the mechanical deformation and stability of the gels’ properties, oscillatory strain sweep tests were performed. Figure 9 shows the changes in the moduli with an increasing deformation amplitude for the hydrogels with different compositions. At moderate strains, G’ > G” was observed for all hydrogels (A/S = 0.5, A/S = 1, and A/S = 2): the elastic component predominated under these conditions. The AM/SPA comonomer ratio had little effect on the nature of the viscoelastic properties of the studied gels, which displayed analogous trends (but different absolute values of G’ and G’’: an increase in SPA decreased G’ and G’’). The results indicated that all the hydrogels in the range of low deformations behaved elastically without changing their microstructure. The linear viscoelastic zone where G’ did not change with deformation extended up to 10% of the deformation amplitude. Up to 1%, G’’ was also practically strain-independent. Between 1 and 10%, the internal friction expressed by G’’ increased steadily (especially in the stiffer gels), but the materials still preserved their dominantly elastic nature (constant G’).

More significant deformations (over 10%) led to a pronounced drop in G’ with a rising strain, as the hydrogel microstructure began to be irreversibly altered. Finally, for high deformations such as 100 or 300% (for A/S = 2 and A/S = 1, respectively), at a critical strain (called the flow point; G’ = G’’), the gel microstructure was destroyed. After that, the hydrogel behaved like a liquid, mainly dissipating energy and not storing it (G’’ > G’) [41]. It was intriguing to note that the flow point of all the studied hydrogels occurred at a strain higher than 100%. In the softest gel, where A/S = 0.5, the flow point was even close to 1000%. Above the flow point, the G’ of the different hydrogels exhibited a steady decrease with the deformation. In some cases, a sample rupture was observed in this final region, like in the case of A/S = 1 (a sudden drop in both moduli).

### 2.4. Chromium(VI) Adsorption Study

**Figure 10:**
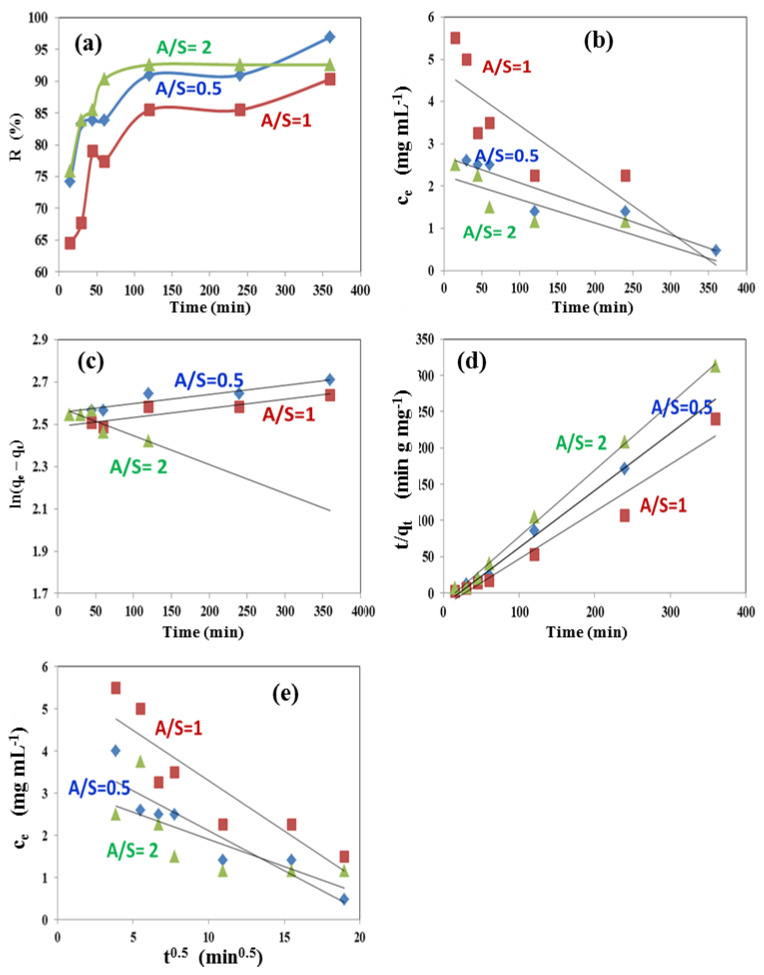
(**a**) Effect of contact time, (**b**) fit (zero-order reaction), (**c**) pseudo-first-order rate, (**d**) pseudo-second-order rate, and (**e**) intra-particle diffusion.

Adsorption tests (see Figure 10a) were performed with potassium chromate (15 mg/L) as a model compound for Cr(IV) pollutants in wastewater. Dried porous hydrogels (20 mg samples) were immersed in this solution (20 mL) in simulated water treatment experiments, and the removal efficiency achieved in one step was found to be between 90 (A/S = 0.5 gel) and 96% (A/S = 2 gel). A higher AM content seemed to favor a more efficient Cr(VI) adsorption. The adsorption was practically complete in 2 h (see Figure 10a), while most of it was achieved already in 1 h. 

Kinetics fits were tested in order to elucidate the mechanism of Cr(VI) adsorption (see Figure 10b–d and fit parameters in Table 4): Pseudo-second-order kinetics best described the process, indicating the importance of coordination bond formation between Cr(IV) and the gel (especially with the AM units that favored more efficient adsorption in Figure 10a). Pseudo-first-order kinetics also yielded a relatively good fit, highlighting the importance of the contact surface area. Intra-particle diffusion analysis according to the Weber–Morris method (Figure 10e) suggested that electrostatic attraction (caused by SPA) accelerated the Cr(VI) adsorption. Thus, the SPA comonomer also played a supportive role in the adsorption of chromate anions.

Generally, a high adsorption ability in combination with the capacity for intense deswelling induced by pH offers promise for potential applications as a regenerable adsorber material for water treatment.

The contact-time dependence of the adsorption efficiency of the A/S = 1, A/S = 0.5, and A/S = 2 gels (Figure 10a) was studied for different times, namely 15, 30, 45, 60, 120, 240, and 360 min, by analyzing the Cr(VI) content in the bath solutions via atomic absorption spectroscopy. As shown in Figure 10a, the affinity of A/S = 1, A/S = 0.5, and A/S = 2 towards Cr(VI) was not the same, and the removal of Cr(VI) by A/S = 1, A/S = 0.5, and A/S = 2 was found to be fast at first, due to the presence of many free functional groups, then became slow, and there was no remarkable increase in the adsorption rate observed after 1 h for A/S = 1, A/S = 0.5, or A/S = 2. The removal efficiency of Cr(VI) with A/S = 1, A/S = 0.5, and A/S = 2 was 96.9, 90.3, and 92.6%, respectively, and it was stable until 6 h. From this finding, we could say that AM more strongly favored Cr(VI) adsorption compared to SPA. This may have been due to the coordination affinity of AM to Cr(VI).

The kinetic fit for the zero-order reaction (Figure 10b) was not suitable for describing the kinetics of Cr(VI) sorption with A/S = 1, A/S = 0.5, and A/S = 2. This kinetics order assumes that increasing the concentration of reactants does not affect the value of the reaction rate [42]. The pseudo-first-order and pseudo-second-order equations (fits: see Figure 10c,d, respectively) were much better-suited to model the kinetics of Cr(VI) adsorption for A/S = 1, A/S = 0.5, and A/S = 2. Concerning the coefficients of correlation (R^2^) presented in Table 2, it can be seen that the pseudo-second-order model provided a better fit for the adsorption data of A/S = 1, A/S = 0.5, and A/S = 2. This kinetics type suggested an important role of the formation of chemical bonds in the adsorption process. The fit for the pseudo-first-order model still appeared suitable for describing the kinetics of Cr(VI) sorption but displayed a somewhat worse correlation. This type of kinetics suggested an important role of surface processes involving the chemisorption and physisorption of Cr(VI) by A/S = 1, A/S = 0.5, and A/S = 2 [3,4,8,13].

The dynamics of Cr(VI) adsorption by the network gels was generally investigated by monitoring the change in the physical dimensions of the hydrogels at different intervals. The pseudo-first- and second-order models provide information about chemical and physical adsorption. The value of *n* in the Peppas and Peppas model provides an indication of the water transport mechanism. The *D* and υ values obtained from Fick’s mechanism and the Peppas and Franson method, respectively, describe the adsorption capacity.

In Figure 10, the pseudo-zero-order fit verified the effect of the concentration, while the first- and second-order fits helped to identify the type of bonds during adsorption.

The analysis of intra-particle diffusion suggested that the studied adsorption was surface adsorption combined with intra-particle diffusion, due to the observation that the straight lines of the fits did not pass through the zero point. This finding was attributed to the strong electrostatic attraction of the Cr(VI) to the A/S = 1, A/S = 0.5, and A/S = 2 surfaces, followed by the diffusion of Cr(VI) into the A/S = 1, A/S = 0.5, and A/S = 2 pores [12]. In the studied gels, the SPA comonomer was responsible for the electrostatic interactions. Hence, SPA was found to also play a supportive role in the adsorption of anionic inorganic pollutants such as Cr(VI), while its main role was to bond cationic pollutants and to tune hydrophilicity.

## 3. Conclusions

Novel pH- and thermo-responsive hydrogels based on hydroxypropyl methyl cellulose (HPMC) grafted with acrylamide (AM) and 3-sulfopropyl acrylate (SPA) were synthesized. The grafted structure was crosslinked to a network by a small amount of a third comonomer (a divinyl compound). This structure was chosen in order to achieve good mechanical properties and accelerated swelling kinetics. HPMC was chosen as a cheap hydrophilic and naturally sourced polymer backbone, while AM and SPA were employed to preferentially bond coordinating and cationic inorganic pollutants, respectively.

Generally, the swelling tendency of the HPMC-g-poly(AM-co-SPA) hydrogels increased with the increasing content of the ionic SPA. The swelling was highest at the graft/comonomer ratio A/S = 0.5, due to the higher hydrophilicity of SPA repeat units compared to AM units.

All the gels displayed a pronounced elastic character, as well as considerably high values of stress at break (several hundred %). The thermal stability was the lowest in the gel with the ratio A/S = 0.5, which was attributed to the highest segmental mobility in this material, while at A/S = 2 the stability was increased by stronger hydrogen bonding (observed by FTIR).

The swelling kinetics of the freeze-dried porous gels indicated that 3 to 5 h were needed for achieving full swelling equilibrium, which appeared to be a reasonable duration in view of the time needed for the efficient adsorption of pollutants from the solution (1 to 2 h) that found itself in the pores of the hydrogels. Generating the porous morphology was important for the potential use as an adsorber, because it offered channels for rapid water transport (upon a stimulus-triggered change in swelling), as well as a large surface area for pollutant adsorption.

Temperature-dependent equilibrium swelling was investigated in the range from 35 to 75 °C and was found to increase with temperature, especially strongly in the case of the ratio A/S = 0.5 (SPA as dominant grafted monomer).

The pH-dependent equilibrium swelling was investigated in the range from 2.0 to 10.0, and a swelling maximum was observed at pH = 7.0 in all gels, while at a more acidic or alkaline pH, the equilibrium swelling decreased. Especially pH-sensitive was the gel with the ratio A/S = 0.5. This behavior was interesting for the potential application as an adsorber, as the cationic or anionic pollutants would first be adsorbed, before being released later by a pH stimulus, which would simultaneously induce the gel to shrink. The considerable pH-sensitivity was attributed purely to ionic strength effects.

Cr(VI) adsorption tests were performed with potassium chromate (15 mg/L) as a model pollutant. Dried porous hydrogels (20 mg sample) were immersed in this solution (20 mL), and the removal efficiency achieved in one step was between 90% (A/S = 0.5 gel) and 96% (A/S = 2 gel) in 1 to 2 h. A higher AM content favored more efficient Cr(VI) adsorption, but kinetics analysis also indicated a supportive kinetic role of the ionic SPA comonomer. The pseudo-second-order (best) and pseudo-first-order (acceptable) kinetics fits proved the dominant role of coordinative bond formation and contact area.

The high removal efficiency achieved for Cr(VI) in combination with the capacity for intense deswelling induced by pH offered promise for potential applications, including as a regenerable adsorber material for water treatment.

## 4. Materials and Methods

### 4.1. Materials

Hydroxypropyl methylcellulose (HPMC), 3-Sulfopropyl acrylate potassium salt (SPA), *N,N′*-methylenebis(acrylamide) (MBA), potassium persulfate (KPS), and potassium chromate were purchased from Sigma Aldrich (Burlington, MA, USA). Acrylamide (AM) was purchased from Fluka (Buchs, Switzerland). All materials were used as received without further purification.

### 4.2. Synthesis of HPMC-g-poly(AM-co-SPA) Hydrogels

The hydrogels were synthesized according to the authors’ previous procedure [3,28]. Initially, 2 g of HPMC was dissolved in 50 mL of distilled water with stirring at 50 °C in a 250 mL flask until a homogeneous solution was formed. Next, the solution temperature was raised to 60 °C, and a calculated amount of KPS (0.12 g) was dissolved in 5 mL of distilled water and added to the HPMC solution. The solution temperature was kept at 65 °C for 10 min to generate free radicals on the HPMC chains. Then, an aqueous solution (10 mL of water) of 6 g of the monomers AM and SPA (ratio 1:1, 1:2, or 2:1 wt/wt) plus 0.24 g of the crosslinking comonomer MBA was added to the HPMC solution. The reaction mixture was briefly stirred at 580 rpm and subsequently left to react at the temperature of 70 °C, which was maintained for 3 h. Next, the resulting hydrogel was washed with distilled water (3 times) and with acetone (3 times) in order to remove unreacted monomers.

The hydrogel was converted to a dry porous form by freezing at −80 °C for 3 h, followed by drying for 3 days, using a Christ Alpha 1–2 LD Plus freeze-dryer (Martin Christ Gefriertrocknungsanlagen GmbH, Osterode am Harz, Germany).

The hydrogels were coded as A/S = 1, A/S = 0.5, and A/S = 2, according to the ratios of the grafted comonomers AM:SPA = 1:1, 1:2, and 2:1 wt/wt, respectively.

### 4.3. Characterization and Analyses

#### 4.3.1. Buffer Solutions with Different pH Values

Solutions for setting several pH values were prepared as follows: The acidic pH 2.0 solution was obtained by mixing 0.1 M HCl (10.6 mL) and 0.1 M KCl (89.4 mL); the pH 4.0 solution was obtained by mixing 0.1 M citric acid (31 mL), 0.1 M sodium citrate (19 mL), and deionized water (50 mL). The pH 7.0, 8.0, and 10.0 solutions were obtained by mixing 0.1 M disodium hydrogen phosphate (75.6, 95.51, and 96.53 mL); 0.1 M HCl (24.4, 4.49, and 0 mL); and 0.1 M NaOH (0, 0, and 3.36 mL).

#### 4.3.2. Swelling Properties

The swelling behavior of the hydrogels was observed by submerging 0.1 g of freeze-dried hydrogel samples in distilled water or buffer solution and incubating them at room temperature for the desired amount of time. The bloated hydrogel was then taken out and weighed after being pushed between two filter sheets in order to remove the extra water. The swelling (sw%) was determined as follows:

(1) $$sw\%=\frac{m_{t}-m_{0}}{m_{0}}\;100\%$$

where ***m_t_*** and ***m*_0_** are the mass of the swollen gel at time ***t*** and the mass of dry hydrogel, respectively [32].

Alternatively, the swelling ratio (Q) is often used in the literature, which is defined as:

(2) $$Q=\frac{m_{sw}}{m_{dry}}$$

where *m_sw_* is the mass of swollen gel before drying, and *m_dry_* is the mass of slowly dried compact non-porous gel. sw% can be converted to Q as follows: Q = (sw%/100) + 1.

#### 4.3.3. Fourier-Transform Infrared Spectroscopy (FT-IR)

Fourier-transform infrared spectra were collected via a Mattson 5000 spectrometer (Pye Unicam, Cambridge, UK) using the KBr disk method.

#### 4.3.4. Scanning Electron Microscopy and EDX Microanalysis

Dried samples of the hydrogels were frozen in liquid nitrogen and gently cut to obtain a smooth surface. The surface morphology of the samples was visualized by a high-resolution FEGSEM microscope (MAIA3, TESCAN, Brno, Czech Republic) equipped with detectors of secondary electrons (SEs) and backscattered electrons (BSEs) and a detector for the energy-dispersive analysis of X-rays (EDX; detector X-MaxN 20; from Oxford Instruments, Abingdon, Oxford, UK). Prior to SEM examination, a conductive thin Pt film was deposited on the samples using a vacuum sputter coater SCD 050 (Leica, Wetzlar, Germany).

#### 4.3.5. Thermogravimetric Analysis (TGA/DTG)

TGA analysis was performed on dried and powdered hydrogel samples using a Perkin Elmer thermogravimetric analyzer by heating the sample to 1000 °C at a rate of 10 °C/min under N2 atmosphere.

The kinetics of thermal decomposition were also evaluated in order to determine the activation energy (*E*) of the thermal degradation. Equations (1) and (2) were applied under the Coats–Redfern approach:

(3) $$log [\frac{1-{\left(1-\alpha \right)}^{1-n}}{{T^{2}(1-n)}^{}}]=log\frac{AR}{\beta E}[1-\frac{2RT}{E}]-\frac{E}{2.303RT}\;\;\;for\;\;n\neq 1$$

(4) $$log [\frac{-\log\left(1-\alpha \right)}{{T^{2}}^{}}]=log\frac{AR}{\beta E} [1-\frac{2RT}{E}]-\frac{E}{2.303RT}\;\;for\;\;n=1$$

where α is the fractional conversion, *n* is the order of degradation, *T* (K) is the temperature, *A* (s^−1^) is the frequency factor, *R* (kJ/mol.K) is the universal gas constant, ß (K/min) is the heating rate, and *E* is the activation energy.

Plotting the relationship between {log_10_ [1 − (1 − α)^1−n^]/*T*^2^ (1 − *n*)} and 1/*T* using the suitable *n* value (order of degradation) should show a straight-line correlation in accordance with Equation (3). For *n* = 1, the relationship between log {−log(1 − α)]/*T*^2^} and 1/*T* is plotted in accordance with Equation (4). To obtain the fit result, the least squares method was used by selecting several *n* values (ranging from 0 to 3.0), calculating the correlation coefficient (r) for each value of *n*, and estimating the standard error (SE). The frequency factor *A* was determined from the intercept (log *AR*/ßE) of the Coats–Redfern equation by the most suitable value of *n*, whilst the activation energy (*E*) was calculated from the slope (*E*/2.303*R*). Equation (5) was used to estimate the other kinetic parameters, such as enthalpy- (∆*H*), entropy- (∆*S*), and free energy change (∆*G*):

(5) $$∆H=E-RT;\;\;\;\;∆G=∆H-T∆S\;\;\;and\;\;\;∆S=2.303\left(log\frac{Ah}{kT}\right)R$$

where *h* and *k* are the Planck and Boltzmann constants, respectively [3,4,8,13].

#### 4.3.6. Shear Modulus of Gels

The shear modulus (*G*) of the prepared gels was measured on ARES-G2 (from TA Instruments, New Castle, DE, USA) between parallel plates in slow uniaxial compression mode at room temperature. The uniaxial compression was increased from 0% to 10% in 2 min, while the applied compression force *F* was recorded. The shear modulus *G* was calculated using the following equation:

*G* = *F*/*S*_0_ (λ^−2^ − λ) (6)

where *S*_0_ is the initial cross-section of the sample before measurement, λ = *l*/*l*_0_, and *l* and *l*_0_ are the compressed and initial height of the sample, respectively. The geometry of the samples was cylindrical, 10 mm in diameter and 10 mm in height [43].

#### 4.3.7. Rheology

The rheological properties of the hydrogels prior to freeze-drying were measured on a strain-controlled ARES-G2 rheometer (TA Instruments, New Castle, DE, USA) using a parallel plate fixture with a diameter of 30 mm (plates with a cross-hatched surface to prevent slipping). Frequency sweep experiments were performed in a frequency range from 0.05 to 100 rad/s at a strain amplitude of 1% and a constant temperature of 25 °C. In addition, the linear viscoelasticity region was determined in view of the dependence of the storage modulus on the strain amplitude, which was measured at 25 °C at a frequency of 1 Hz.

#### 4.3.8. Swelling Kinetics Models Used in this Study

Considering the first-order kinetics (*K*_1_ as rate constant), the swelling rate at any time may be expressed using the equation:

(7) $$ln\frac{W_{e1}}{W_{e1}-W_{t}}=K_{1}t$$

In the case of second-order kinetics (*K*_2_ as rate constant), the following equation holds:

(8) $$\frac{t}{W_{t}}=\frac{1}{K_{2}W_{e2}^{2}}+\frac{1}{W_{e2}}t$$

where *W_e_*_1_ and *W_e_*_2_ stand for the quantities of water absorbed by the gel at equilibrium for the pseudo-first- and second-order models, respectively [44].

Peppas and Peppas described simple water-transport phenomena in hydrogel networks using the following equation (below 0.6, fractional swelling values):

(9) $$ln\frac{W_{t}}{W_{e}}=K\;t^{n}$$

where *W_t_* and *W_e_* stand for the quantities of water absorbed by the gel in time ‘*t*’ and at equilibrium, respectively.

A plot of $ln\frac{W_{t}}{W_{e}}$ versus *ln t* was used to calculate *n* and *K*, from the slope and intercept, respectively. *K* is the swelling constant characteristic of the system under consideration; and *n* represents the diffusion exponent that throws light on the mode of water transport into the gel.

Based on Fick’s law, a mutual diffusion coefficient (*D*) can be calculated as follows:

(10) $$ln\frac{W_{t}}{W_{e}}=\left(\frac{4}{\pi^{0.5}}\right)(\frac{D\;t}{L^{2}}{)}^{n}$$

The Peppas and Franson method was followed to estimate the water penetration velocity (υ) of the hydrogels:

(11) $$\upsilon =\left(\frac{dW_{t}}{dW_{e}}\right)\left(\frac{1}{\rho }\right)\left(\frac{1}{2A}\right)$$

where *ρ* is the density of water (or of the aqueous solution) and 2*A* is the surface area of the hydrogel [34,35].

#### 4.3.9. Cr(VI) Adsorption Study

The Cr(VI) adsorption experiments were performed by placing a dry adsorber gel (mass: 20 mg) into 20 mL of a potassium chromate solution, whose initial concentration was 15 mg/L. The solution with the adsorber was stirred at room temperature for different time intervals (15, 30, 45, 60, 120, 240, and 360 min) in order to study the adsorption kinetics.

The final residual concentrations of Cr(VI) in the bath solution were measured using a PerkinElmer atomic absorption spectrophotometer, Model 3110, from PerkinElmer, Waltham, MA, USA.

Evaluation and fitting of adsorption kinetics:

To characterize the sorption efficiency of the prepared HPMC-g-poly(AM-co-SPA) hydrogels for removing Cr(VI) from water, the comparative removal efficiency (*R*%) and adsorption capacity (*q_e_*) of the HPMC-g-poly(AM-co-SPA) hydrogels were calculated at different contact times using the equations:

(12) $$R\%=\frac{C_{0}-C_{t}}{C_{0}}\;100\%$$

(13) $$q_{e}=\frac{C_{0}-C_{t}}{m}\;V$$

where *C*_0_ and *C_t_* are the Cr(VI) concentrations (mg/L) in the solution before and after adsorption; *V* is the volume of the solution (L); and *m* is the amount (g) of sorbent employed in the adsorption experiment [3,4,8,12,45].

The zero-order, pseudo-first-order, and pseudo-second-order models could be determined from the equations:

(14) $$C_{e}=C_{0}-K\;t$$

(15) $$\ln [q_{e}-q_{t}]=\ln q_{e}-K_{1}t$$

(16) $$\frac{t}{q_{t}}=\frac{1}{K_{2}q_{e}^{2}}-\frac{t}{q_{t}}$$

where *q_e_* and *q_t_* are the amounts of Cr(VI) adsorbed (mg/g) at equilibrium sorption capacity and time *t*, respectively; *C_e_* is the final concentration of the HPMC-g-poly(AM-co-SPA)adsorbate with *t* (contact time); *K*_1_ (min^−1^) is the pseudo-first-order rate constant of adsorption; and *K*_2_ is the rate constant of pseudo-second-order adsorption. Values of *q_e_*^2^ and *K*_2_ were calculated from the slope and intercept of the plot of *t*/*q_t_* against *t*, respectively [12,34,38].

The Weber–Morris intra-particle diffusion could be determined from the equation:

*q t* = *K*_3_
*t*^1/2^ + *C* (17)

where *K*_3_ is the intra-particle diffusion rate constant, and *C* is the slope that represents the thickness of the boundary layer [12].

## Figures and Tables

**Table 1:** Mean hydrogen bond strength (MHBS) of HPMC-g-poly(AM-co-SPA) hydrogels.

Hydrogel	A/S = 0.5	A/S = 1	A/S = 2
MHBS	0.98	0.96	0.98

**Table 2:** TGA results and thermodynamic and kinetic parameters of the hydrogels’ degradation in N_2_.

Sample	Stage	TGA Range, (°C)	DTG Peak (°C)	Mass Loss (%)	*n* ( )	R^2^ ( )	A (s^−1^)	∆H(kJ mol^−1^)	∆S(kJ mol^−1^)	∆G(kJ mol^−1^)	SE ( )	*E*a, (kJ mol^−1^)
**A/S = 1**	**1st** **2nd** **3rd**	30.53–189.40 222.64–416.45 491.67–751.95	71.49 384.72 433.26	3.20 42.33 14.61 **∑ML%= 57.14** **∑RW%= 42.86**	1.5 1.5 2.0	0.9788 0.9691 0.9994	0.33 0.28 0.29	14.60 24.29 11.35	−0.25 −0.26 −0.26	102.50 196.62 196.57	55 × 10^−2^ 73 × 10^−2^ 30 × 10^−3^	17.46 29.76 17.22 **∑E = 64.44**
**A/S = 0.5**	**1st** **2nd** **3rd**	39.35–130.88 178.98–400.19 408.87–759.71	92.34 379.12 421.20	4.74 36.54 21.15 **∑ML%= 62.43** **∑RW%= 37.57**	1.5 1.5 3.0	0.9641 0.9361 0.9997	0.32 0.23 4.59	14.88 21.75 9.86	−0.25 −0.26 −0.23	108.41 192.50 175.98	61 × 10^−2^ 12 × 10^−1^ 30 × 10^−3^	17.92 27.17 15.63 **∑E = 60.72**
**A/S = 2**	**1st** **2nd** **3rd**	39.83–195.09 229.55–411.60 429.65–759.70	102.66 380.83 443.52	4.06 41.37 21.91 **∑ML%= 67.34** **∑RW%= 32.66**	1.5 1.5 3.0	0.9854 0.9885 0.9993	0.30 0.26 4.51	18.88 29.55 9.98	−0.25 −0.26 −0.23	120.66 201.21 181.73	23 × 10^−2^ 22 × 10^−1^ 47 × 10^−3^	22.17 34.99 15.94 **∑E = 73.10**

**Table 3:** Experimental swelling (We, exp. (g/g)) and model swelling (% We 1, calc. (g/g); We 2, calc. (g/g)) of HPMC-g-poly(AM-co-SPA) hydrogels.

Kinetic Model	Parameter	A/S = 1	A/S = 0.5	A/S = 2
**Pseudo-first-order**	**W_e; exp._ (g/g)**	28.29	69.43	40.83
	**W_e 1; calc._ (g/g)**	0.99	0.98	0.95
	**K_1_**	0.001	0.006	0.004
	**R^2^**	0.9274	0.9180	0.9554
**Pseudo-second-order**	**W_e 2; calc._ (g/g)**	28.61	69.55	42.82
	**K_2_**	0.004	0.008	0.009
	**R^2^**	0.7913	0.8483	0.8661
**Peppas and Peppas**	**K_3_**	3.303	3.708	3.122
	***n***	0.215	0.561	0.463
	**R^2^**	0.9226	0.9096	0.9602
**Fick’s diffusion mechanism**	**D**	0.027	0.042	0.041
	**R^2^**	0.9202	0.9385	0.9083
**Peppas and Franson method**	υ	0.0010	0.00137	0.00135
	**R^2^**	0.9850	0.9472	0.9135

**Table 4:** Comparison between the estimated adsorption rate constants, rate constants, and correlation coefficients associated with the pseudo-first-order and pseudo-second-order rates and intra-particle diffusion.

Kinetic Model	Parameter	A/S = 1	A/S = 0.5	A/S = 2
Zero-order reaction	K_0_	77 × 10^−3^	94 × 10^−3^	50 × 10^−3^
	R^2^	0.8951	0.6288	0.6122
Pseudo-first-order	q_exp._ (mg/g)	15.01	13.98	14.33
	q_Calc._ (mg/g)	12.40	10.96	12.61
	K_1_	57 × 10^−4^	78 × 10^−4^	45 × 10^−4^
	R^2^	0.8568	0.8205	0.7729
Pseudo-second-order	q_Calc._ (mg/g)	0.51	1.52	1.09
	K_2_	22 × 10^−2^	35 × 10^−2^	65 × 10^−2^
	R^2^	0.9933	0.9606	0.9975
Intra-particle diffusion	K_p_ (mg·g^−1^·min^−1^(0.5))	0.18	0.23	0.12
	C (mg/g)	4.00	5.68	3.19
	R^2^	0.8569	0.8031	0.5332

## Data Availability

Data are contained within the article.
